# Genomic profiling, prognosis, and potential interventional targets in young and old patients with cholangiocarcinoma

**DOI:** 10.1080/15384047.2023.2223375

**Published:** 2023-06-19

**Authors:** Junhua Wang, Yaoting Shi, Jianbo Chen, Juying Liu, Xiaotian Zhao, Jiaohui Pang, Ximin Sun, Yichen Tian, Qiuxiang Ou, Feng Xia, Yunjie Chen

**Affiliations:** a Department of Biliary-Pancreatic Surgery, The First People’s Hospital of Foshan, Foshan, China; b Department of Medical Oncology, Beidahuang Industry Group General Hospital, Harbin, China; c Department of Medical Oncology, The First Affiliated Hospital of Xiamen University, Xiamen, China; d School of Medicine, Xiamen University, Xiamen, China; e Department of Radiation Oncology, Jiangsu Cancer Hospital & Jiangsu Institute of Cancer Research & the Affiliated Cancer Hospital of Nanjing Medical University, Nanjing, China; f Geneseeq Research Institute, Nanjing Geneseeq Technology Inc, Nanjing, China; g Department of Hepatobiliary Surgery, The First Hospital Affiliated to AMU (Southwest Hospital), Chongqing, China; h Department of Hepatopancreatobiliary Surgery, Hwa Mei Hospital, University of Chinese Academy of Sciences (Ningbo No. 2 Hospital), Ningbo, China

**Keywords:** Genomic profiling, prognosis, patient age, cholangiocarcinoma

## Abstract

Molecular mechanisms behind potentially inferior prognosis of old cholangiocarcinoma (CCA) patients are unclear. Prevalence of interventional targets and the difference between young and old CCA patients are valuable for promising precision medicine. A total of 188 CCA patients with baseline tumor tissue samples were subgrouped into the young (≤45 years) and old (>45 years) sub-cohorts. Somatic and germline mutation profiles, differentially enriched genetic alterations, and actionable genetic alterations were compared. An external dataset was used for the validation of molecular features and the comparison of overall survival (OS). Compared to young patients, *KRAS* alterations were more common in old patients (*P* = .04), while *FGFR2* fusions were less frequent (*P* = .05). *TERT* promoter mutations were exclusively detected in old patients. The external dataset (*N* = 392) revealed no significant difference in OS between young and old patients; however, old patient-enriched *KRAS* (hazard ratio [HR]: 1.96, 95% confidence interval [CI]: 1.37–2.80) and *TERT* alterations (HR: 2.03, 95% CI: 1.22–3.38) were associated with inferior OS. Approximately 38.3% of patients were identified of actionable oncogenic mutations indicative of a potential response to targeted therapy or immunotherapy. Actionable *FGFR2* fusions (*P* = .01) and *BRAF^V600E^* (*P* = .04) mutations were more frequent in young females than old patients. The enrichment of *KRAS*/*TERT* alterations in CCA patients over 45 years resulted in inferior OS. Approximately one-third of CCA patients were eligible for targeted therapy or immunotherapy given the actionable mutations carried, especially young females.

## Introduction

Cholangiocarcinoma (CCA), with three subtypes including intrahepatic cholangiocarcinoma (iCCA), perihilar cholangiocarcinoma (pCCA) and distal cholangiocarcinoma (dCCA),^1^ is the second most common primary hepatic malignancy, accounting for approximately 15% primary liver tumors^2^. pCCA and dCCA are also collectively referred to as extrahepatic CCA (eCCA)^3^. Several well-known risk factors for CCA have been identified, such as alcohol consumption, smoking, hepatitis B/C virus infection, genetic alterations related to DNA repair, and the potential influence of obesity as well as drugs.^4,5^ CCA patients are usually asymptomatic in early stage,^6,7^ resulting disease being diagnosed in advanced stage and a poor prognosis. For CCA patients eligible for resection treatment followed by adjuvant chemotherapy, their median overall survival (mOS) and relapse-free survival (mRFS) are 51.1 and 24.4 months, respectively,^8^ with a high relapse rate.^9,10^ On the other hand, the expected mOS and median progression-free survival (mPFS) are 11.7 and 8.0 months, respectively, for CCA patients receiving palliative systemic chemotherapy due to unresectable disease.^8^

The influence of patient age on CCA prognosis is inconsistent across various studies, with unclear potential molecular mechanisms behind. Several previous studies have reported the negative association between patient age and clinical outcomes. In the United States, old iCCA patients ≥45 years had worse 5-year survival rates when compared to young patients under 45.^11^ One study focused on iCCA demonstrated that old patients had significantly inferior OS than young patients, and old age was identified as an independent prognostic factor in multivariate analyses.^12^ On the other hand, in a Canadian study including 200 iCCA and 191 pCCA patients, it has been revealed that similar survival benefits from surgery and palliative chemotherapy were observed irrespective of patient age.^13^ Similar results were reported in the sub-analysis of 13 prospective trials on advanced biliary cancer receiving palliative chemotherapy.^14^ A number of previous sequencing studies have been completed, discovering the hotspot *IDH* mutation in iCCA, identifying the actionable *FGFR2* fusion mutation, as well as emphasizing the genomic complexity of CCA across subtypes.^15–17^ Nevertheless, few research works have comprehensively investigated genetic alterations in old and young CCA patients separately.

Molecular profiling of tumor tissue samples is able to guide the development of treatment option for CCA patients with advanced diseases, even though chemotherapy is currently recommended as the standard of care. The ABC−02 trial reported that patients with locally advanced or metastatic CCA, gallbladder cancer, or ampullary cancer receiving the combination of cisplatin and gemcitabine as the first-line palliative chemotherapy achieved significant survival advantage, compared to those treated with gemcitabine alone.^18^ After disease progression on first-line chemotherapy, the addition of FOLFOX (folinic acid, 5-FU and oxaliplatin) to active symptom control is recommended as the standard of care for second-line treatment, owing to superior OS as well as increased 6-month and 12-month OS rates.^19^ In addition to chemotherapy, targeted therapies could be options for CCA patients with locally advanced or metastatic disease, such as pemigatinib and infigratinib for patients with *FGFR2* fusion or other rearrangement,^20,21^ ivosidenib for previously treated patients carrying *IDH−1* mutations,^22^ etc. Immunotherapy might be another effective treatment option, while a subsequent trial was unable to confirm the efficacy of pembrolizumab in biliary tract carcinoma (BTC), with a responding rate as low as 6%.^23,24^ Therefore, it is meaningful to well understand the proportion of CCA patients harboring each potential intervention target and the differences between age groups.

Herein, this research aimed to comprehensively studied somatic and germline mutation profiles, as well as differentially expressed genes between young and old CCA patients. CCA prognosis data from one external data set were then explored. Prevalence of actionable mutations in young and old CCA patients was also investigated separately.

## Material and methods

### Patients

Participants were retrospectively included from the database of Nanjing Geneseeq Technology Inc., between August 2016 and December 2020. Main inclusion criteria were: 1) adults ≥18 years old; 2) with pathologically confirmed CCA; 3) with baseline tumor tissue samples within 90 days after initial diagnosis and prior to systemic treatment. TNM stages in CCA were determined according to the 8^th^ edition of the American Joint Committee on Cancer classification. Demographics and clinical characteristics of participants, including age, gender, treatment history, the location of CCA, and family history of BTC and/or hepatocellular carcinoma (HCC), were obtained from the database of Nanjing Geneseeq Technology Inc. According to a previous study focused on a Chinese cohort in which a cutoff value of 45 years was used to identified young CCA patients,^25^ our patients whose ages at initial diagnosis ≤45 years were grouped into the young subgroup, and patients over 45 years at initial diagnosis were grouped into the old subgroup. The procedures of this study were approved by the Medical Ethics Committee of Nanjing Geneseeq Medical Laboratory (NSJB-MEC−2022-02), and each patient provided written informed consent.

### DNA extraction, library preparation, and NGS data processing

Genomic profiling of baseline tumor tissue samples was performed using targeted next-generation sequencing (NGS) covering 425 cancer-related genes at a centralized Clinical Laboratory Improvement Amendments-certified, College of American Pathologists-accredited clinical laboratory (Nanjing Geneseeq Technology Inc). Genomic DNA from baseline formalin-fixed paraffin-embedded (FFPE) samples was extracted with QIAamp DNA FFPE Tissue Kit (QIAGEN); genomic DNA from leukocyte controls was extracted using DNeasy Blood and Tissue Kit (QIAGEN), from peripheral blood centrifuged at 1,800× g for 10 min at room temperature within 2 h after collection. Sequencing libraries were prepared using KAPA Hyper Prep Kit (KAPA Biosystems), and targeted enrichment was performed with customized xGen lockdown probes panel (Integrated DNA Technologies), Human cot−1 DNA (Life Technologies) and xGen Universal blocking oligos (Integrated DNA Technologies). Libraries were sequenced on Illumina Hiseq NGS platforms (Illumina). All procedures were conducted following the manufacturers’ instructions.

FASTQ file quality was controlled using Trimmomatic, removing leading/trailing low-quality (reading <15) or N bases.^26^ Sequencing data were then aligned to the reference human genome (build hg19) and processed using the Picard suite and the Genome Analysis Toolkit (GATK).^27,28^ A somatic mutation, filtered for common single nucleotide polymorphisms and germline mutations, was retained when it had at least 0.5% mutant allele frequency, at least three unique reads on different strands with good quality scores, and not present in public databases (Exome Variant Server, 1000 Genomes Project, and Exome Aggregation Consortium) at a population frequency > 1%. Gene fusions and copy number variations (CNV) were analyzed using FACTERA and ADTEx, respectively.^29,30^ and manually reviewed in Integrative Genomics Viewer Software (IGV, Broad Institute). The cutoffs of retaining CNV were 1.6 for CNV gain and 0.6 for CNV loss.

### Statistical analysis

Fisher’s exact test and two-sample t-test were performed to compare the frequencies and means of variables between young and old CCA patients, respectively. For survival data, Kaplan-Meier curves for OS were generated, and log-rank tests were used to compare differences. Cox proportional hazards models were fitted to estimate hazard ratios (HR) and 95% confidence intervals (CI), and the proportionality of hazards was assessed using log(−log) survival plots. Data were analyzed using R software (version 4.0.3), and the *survival* package. Differential enrichment analysis for signaling pathways in which altered genes were located was conducted using KEGG pathway enrichment analysis. All quoted *P*-values were two-tailed, with values less than 0.05 considered to be statistically significant.

## Results

### Patient overview

**Figure 1.**
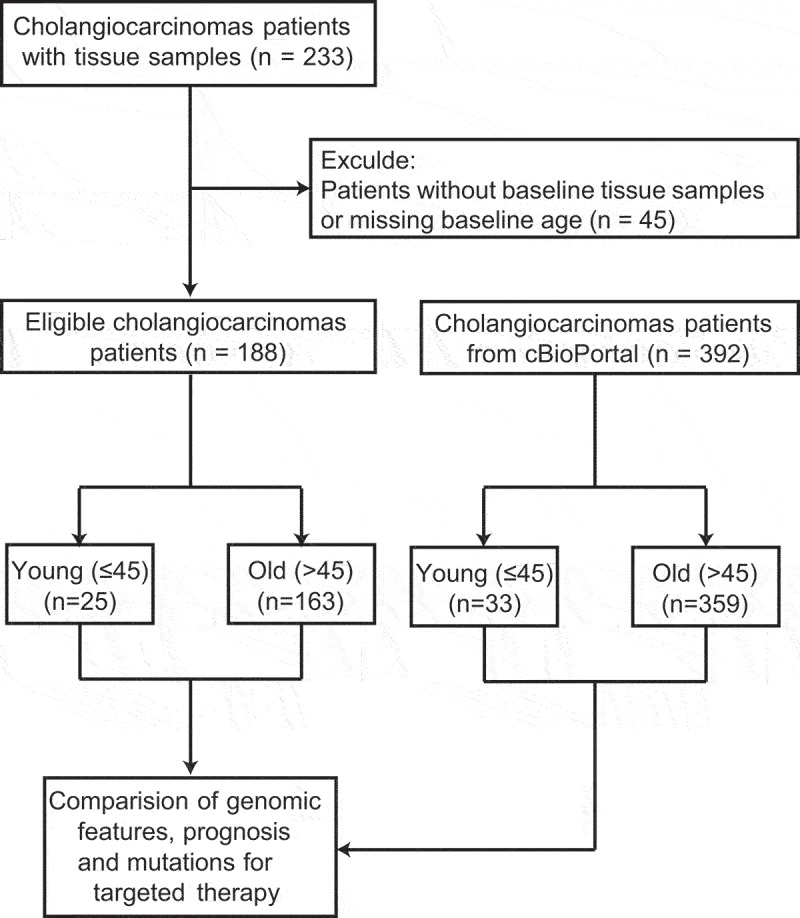
The flowchart of enrollment and analyzable patients.

A total of 188 eligible CCA patients with baseline tumor tissue samples were enrolled in this study, including 25 young patients ≤45 years and 163 old patients over 45 years (Figure 1). The median age of the entire study cohort was 60 (range: 23–81) years, and 56% (105/188) patients were males (Table 1). At initial diagnosis, 35% (65/188) and 19% (36/188) patients were diagnosed with stage IV and stage I–III disease, respectively; however, other 46% (87/188) patients had missing data for clinical stage. Within the panel covering 425 genes, 33% (62/188) patients were detected with tumor mutational burden (TMB) of at least 6 muts/Mb. Although treatment history records were missing in 81% (152/188) participants, it presented as a majority of patients treated with chemotherapy alone (15%, 28/188). The remaining eight patients had ever received targeted therapy (2%, 4/188) or immunotherapy (2%, 4/188). Family history of BTC/HCC were only identified in 3% patients (6/188), while patients with missing values accounted for 18% of the study cohort. The median ages of the young and old subgroups were 39 (range: 23–44) and 61 (range: 46–81) years, respectively (Table 1). Compared to the young subgroup, female patients appeared to be less frequently observed in the old subgroup (42% *vs*. 60%, *P* = .13); however, the proportions of patients diagnosed with stage I–III disease were similar between two subgroups (19% *vs*. 20%, *P* > .99). Of note, a positive relationship between patient age and TMB level at initial diagnosis was observed (≥6 muts/Mb: 36% *vs*. 12%, *P* = .02).

A total of 188 cholangiocarcinoma patients were included in the study cohort, including 25 young patients and 163 old patients. From an external data set, 392 patients (33 young and 359 old patients) with baseline genetic alteration data and overall survival data were identified.

**Table 1.** Demographics and clinical characteristics of patients.

Characteristics	Overall (*n* = 188)	Young (*n* = 25)	Old (*n* = 163)	*P* value
Age at initial diagnosis, median (range), y	60 (23–81)	39 (23–44)	61 (46–81)	<0.001*
Gender, No. (%)				0.13
Female	83 (44)	15 (60)	68 (42)	
Male	105 (56)	10 (40)	95 (58)	
Clinical stage at initial diagnosis, No. (%)				>0.99
I – III	36 (19)	5 (20)	31 (19)	
IV	65 (35)	10 (40)	55 (34)	
Unknown	87 (46)	10 (40)	77 (47)	
TMB, No. (%)				0.02*
<6 muts/Mb	126 (67)	22 (88)	104 (64)	
≥6 muts/Mb	62 (33)	3 (12)	59 (36)	
Treatment, No. (%)				>0.99
Chemotherapy	28 (15)	5 (20)	23 (14)	
Targeted therapy	4 (2)	1 (4)	3 (2)	
Immunotherapy	4 (2)	1 (4)	3 (2)	
Unknown	152 (81)	18 (72)	134 (82)	
Family history of BTC/HCC, No. (%)				0.18
With	6 (3)	2 (8)	4 (2)	
Without	149 (79)	18 (72)	131 (80)	
Unknown	33 (18)	5 (20)	28 (17)	

Abbreviations: TMB, Tumor mutational burden; BTC, biliary tract carcinoma; HCC, hepatocellular carcinoma.

*Statistically significant

### Distinctive mutation landscapes and development mechanisms related to patient age

**Figure 2.**
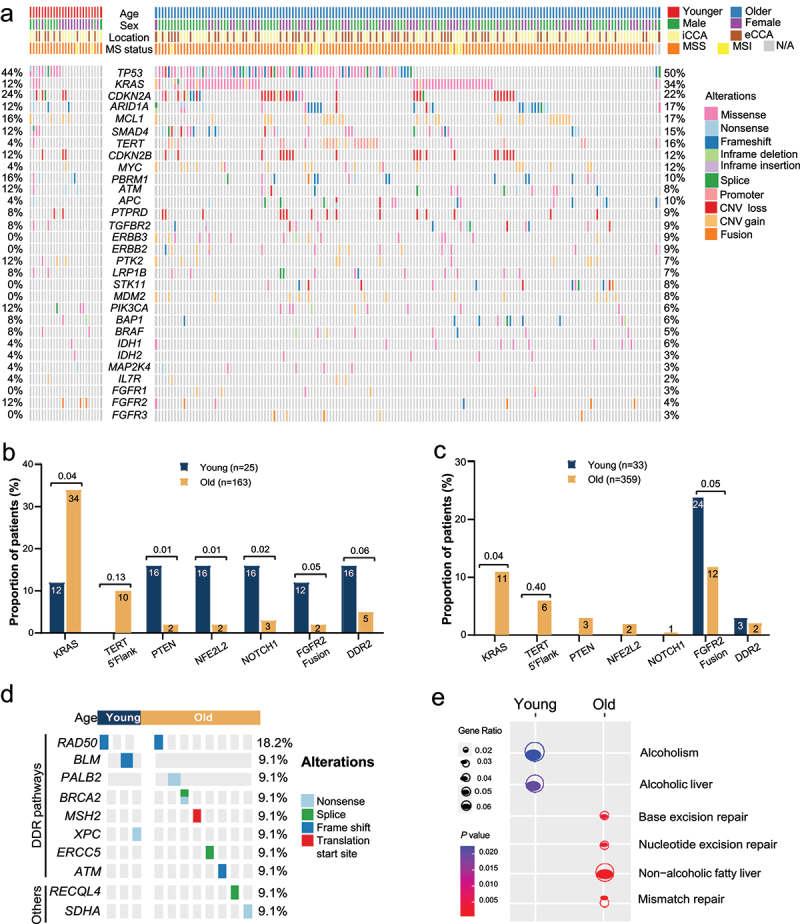
Baseline genomic profiles of young and old patients with cholangiocarcinoma.

For somatic alterations, the most common altered gene in the entire study cohort was *TP53* (49%, 92/188), detected in 44% young patients and 50% old patients. Other frequently mutated genes included *KRAS* (31%, 59/188), *CDKN2A* (22%, 42/188), and *ARID1A* (16%, 31/188). We also observed CNV gain alterations of *MCL1*, *MYC*, *PTK2*, and *MDM2*, as well as CNV loss alterations of *CDKN2A*, *CDKN2B*, and *PTPRD* (Figure 2a). Compared to 25 young patients, mutated *KRAS* genes were more frequently identified in old patients (34% *vs*. 12%, *P* = .04), and *TERT* promoter mutations were exclusively detected in old patients (10% *vs*. 0%, *P* = .13) (Figure 2b). However, altered *PTEN* (16% *vs*. 2%, *P* = .01), *NFE2L2* (16% *vs*. 2%, *P* = .01), and *NOTCH1* (16% *vs*. 3%, *P* = .02) genes were enriched in the young subgroup. Notably, *FGFR2* fusions, one type of CCA actionable mutations, were also more frequently detected in young patients than old patients (12% *vs*. 2%, *P* = .05). In addition, the prevalence of *DDR2* alterations appeared to be relatively high in young patients in comparison to old patients (16% *vs*. 5%, *P* = .06). When compared iCCA with eCCA patients’ samples, *TP53* (57% *vs*. 43%, *P* = .04) and *SMAD4* mutations (21% *vs*. 12%, *P* = .02) were more prevalent in eCCA, whereas *IDH1* (9% *vs*. 3%, *P* = .05) mutations and *FGFR2* fusions (6% *vs*. 0%, *P* = .04) appeared to be more common in iCCA.

(a) The genomic profiles of 188 patients in the study cohort. (b) Somatic alterations with different prevalence between young and old patients in the study cohort. (c) Somatic alterations with different prevalence between young and old patients in the external cohort. (d) Germline alterations of patients in the study cohort. (e) KEGG pathway enrichment analysis for altered genes.

From one bi-institutional study,^31^ a total of 392 iCCA patients with at least one somatic alteration and available OS data were identified for the validation of genomic features and the exploration of prognosis. Thirty-three patients aged ≤45 years were included in the young subgroup, and the remaining 359 over−45-year-old patients were included in the old subgroup. Genomic features and OS data of patients from the external data set were downloaded from cBioPortal for Cancer Genomics (https://www.cbioportal.org/study/summary?id=ihch_msk_2021). Intriguingly, in this external cohort, the proportion of patients with TMB level ≥ 6 muts/Mb was only 11%, and no significant association between patient age and TMB level was observed (Table S1). Although no *KRAS* alteration was detected in the young subgroup, the prevalence of *KRAS* alterations in old patients was only 11%, being much lower than in our study cohort (34%) (Figure 2c). *TERT* promoter mutations were exclusively detected in old patients, which was consistent with the finding in our study cohort. Interestingly, none of *PTEN*, *NFE2L2*, and *NOTCH1* alterations were detected in the young subgroup of the external cohort, while the proportion of old patients carrying altered *PTEN* (3%), *NFE2L2* (2%), and *NOTCH1* (1%) were similar to our study cohort. The association between patient age and *FGFR2* fusions detection remained significant; however, the prevalence of *FGFR2* fusion in this external cohort was higher than that in our study cohort (13% *vs*. 3%, *P* < .001).

Germline mutations were identified in three (12%) young patients and eight (5%) old patients in our study cohort. Except *RECQL4* and *SDHA*, other mutated genes were all located in the DNA damage response pathway (Figure 2d). KEGG analysis demonstrated that alcoholism and alcoholic liver relevant genes were frequently altered among young patients; however, genetic alterations related to nonalcoholic fatty liver and gene repair were enriched in the old subgroup (Figure 2e). The result of KEGG analysis could potentially reveal distinctive risk factors for CCA in young and old people.

### Inferior overall survival due to genetic alterations rather than patient age

**Figure 3.**
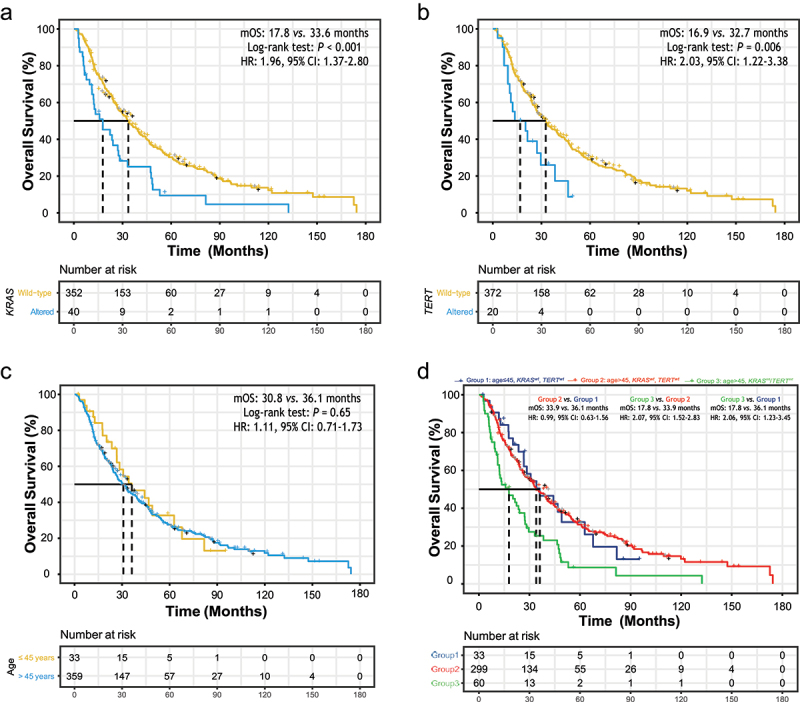
The association of overall survival (OS) with baseline clinical and genetic characteristics.

The mOS of the external cohort was 32.0 (95% CI: 26.8–36.6) months. Compared to patients with wide-type *KRAS* gene, patients harboring altered *KRAS* genes had significantly inferior OS (mOS: 17.8 *vs*. 33.6 months, HR: 1.96, 95% CI: 1.37–2.80, Figure 3a). Similar results were obtained in patients with mutated *TERT* genes (mOS: 16.9 *vs*. 32.7 months, HR: 2.03, 95% CI: 1.22–3.38, Figure 3b). We were not able to observe significant differences in OS across patients with different *FGFR2* mutation status. The mOS of patients with *FGFR2* fusions, with other types of *FGFR2* alterations, and without *FGFR2* alteration were 48.2, 23.8, and 30.7 months respectively. The hazard of death was 14% lower in CCA patients harboring *FGFR2* fusion when compared to those without *FGFR2* alteration, whereas the 95% CI (0.59–1.27) covered 1 (Figure S1a). Similarly, there was no significantly different survival outcomes between patients with *IDH1* mutations and without (mOS: 35.6 *vs*. 30.8 months, HR: 0.81, 95% CI: 0.61–1.09, Figure S1b). *TP53*-mutated CCA patients displayed significantly poorer OS than those with wild-type *TP53* genes (mOS: 13.7 *vs*. 36.6 months, HR: 2.23, 95% CI: 1.67–2.99, Figure S1c). Furthermore, a trend was observed that higher TMB level might be associated with poorer OS (mOS: 26.7 *vs*. 34.1 months, HR: 1.25, 95% CI: 0.98–1.60, Figure S1d).

(a) Altered *KRAS* gene was associated with inferior OS. Median OS (mOS): 17.8 vs. 33.6 months, hazard ratio (HR): 1.96, 95% confidence interval (CI): 1.37–2.80. (b) Altered *TERT* gene was associated with inferior OS. mOS: 16.9 vs. 32.7 months, HR: 2.03, 95% CI: 1.22–3.38. (c) Older patient age was not significantly associated with OS. mOS: 30.8 vs. 36.1 months, HR: 1.11, 95% CI: 0.71–1.73. (d) Old patients without altered *KRAS* or *TERT* mutations had similar OS in comparison with young patients without *KRAS* or *TERT* mutations, while old patients harboring *KRAS* or *TERT* alterations had inferior OS (mOS: 17.8 vs. 33.9 months, HR: 2.07, 95% CI: 1.52–2.83; mOS: 17.8 vs. 36.1 months, HR: 2.06, 95% CI: 1.23–3.45).

We then tried confirming the previously reported association between patient age and OS. The mOS of 359 old patients and 33 young patients were 30.8 (95% CI: 26.5–36.6) months and 36.1 (95% CI: 26.7–67.8) months, respectively, without significant difference in OS between two subgroups (HR: 1.11, 95% CI: 0.71–1.73, Figure 3c). *KRAS* and *TERT* alterations, both enriched in old patients and associated with inferior OS, were potential confounders or effect modifiers of the relationship between patient age and OS. Thus, patients were further grouped according to their ages and *KRAS*/*TERT* gene alterations, and stratified analyses were conducted. For patients without altered *KRAS*/*TERT* genes, old patients (Group 2) display similar OS to young patients (Group 1) (mOS: 33.9 *vs*. 36.1 months, HR: 0.99, 95% CI: 0.63–1.56, Figure 3d). The association of *KRAS*/*TERT* alterations with OS remained significant when old patient with altered *KRAS*/*TERT* genes (Group 3) were compared to those without (Group 2) (mOS: 17.8 *vs*. 33.9 months, HR: 2.07, 95% CI: 1.52–2.83). As expected, Group 3 showed inferior OS than Group1 (mOS: 17.8 *vs*. 36.1 months, HR: 2.06, 95% CI: 1.23–3.45).

### Therapeutic implications of patient’s actionable genomic alterations

**Figure 4.**
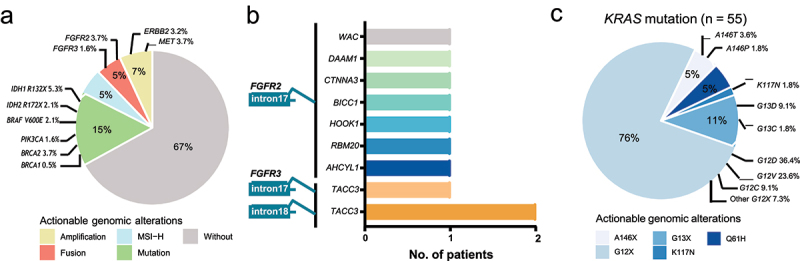
The prevalence of potentially actionable genetic alterations.

Of 188 individuals in our study cohort, 72 (38.3%) patients were potentially eligible for receiving targeted therapy or immunotherapy, and their actionable alterations or immune-related genetic features were summarized in Table 2. Potential intervention targets mainly included *IDH1/2* (7.4%), *BRAF*^*V600E*^ (2.1%), *PIK3CA* (1.6%) and *BRCA1/2* (4.2%) mutations, *ERBB2* (3.2%) and *MET* (3.7%) CNV gains, *FGFR2/3* fusions (5.3%), and microsatellite instability-high (5.3%) (Figure 4a). All the breakpoints of seven patients carrying *FGFR2* fusions were located in *FGFR2* intron 17, and the passenger fusion gene of each *FGFR2* fusion was unique (Figure 4b). In addition to six passenger fusion genes having been reported, including *WAC*, *CTNNA3*, *BICC1*, *HOOK1*, *RBM20*, and *AHCYL1*, a novel passenger gene, *DAAM1*, was observed in our study cohort. *FGFR3* fusion were observed in another three patients, with a same passenger fusion gene of *TACC3*, while breakpoints included *FGFR3* intron 17 and intron 18. Notably, a total of 55 patients had *KRAS* mutations, and 76% patients with *KRAS*^*G12X*^ mutations, while *KRAS*^*G12C*^ mutations were identified in only five patients (Figure 4c), suggesting the motivation of developing treatments to tackle *KRAS* mutations other than *KRAS*^*G12C*^ inhibitors.

(a) The prevalence of potentially actionable genetic alterations. Approximately 33% of 188 cholangiocarcinoma patients in our study cohort were eligible for targeted therapy. (b) Seven patients carried *FGFR2* fusion mutations, with all breakpoints in *FGFR2* intron 17, and a novel passenger gene, *DAAM1*, was detected. *FGFR3* fusion were observed in another three patients, with a same passenger fusion gene of *TACC3*. (c) *KRAS* mutations were identified in 55 of 188 patients, while *KRAS^G12C^* mutations only accounted for 9% cases.

**Table 2.** Actionable genomic features in cholangiocarcinoma.

Biomarkers	No. patient	Percentage (%)
*IDH1/2* Mutation	14	7.4
*FGFR1–4* Fusion	10	5.3
*BRCA1/2* Mutation	8	4.3
*MET* CNV Gain	7	3.7
*ERBB2* CNV Gain	6	3.2
*KRAS G12C*	5	2.7
MSI-High	10	5.3
TMB-High (>10 muts/Mb)	27	14.4
Total	72	38.3

Abbreviations: MSI, microsatellite instability; TMB, Tumor mutational burden.

The proportions of patients with the potential of receiving targeted therapy were similar between young and old patients (36% *vs*. 27%, *P* = .35, Figure S2a). Females were more likely to be eligible for targeted therapy irrespective of their ages (young subgroup: 60% *vs*. 0%, *P* = .002; old subgroup: 37% *vs*. 20%, *P* = .02). Treatments targeting *FGFR2* fusions (20% *vs*. 2%, *P* = .01) and *BRAF*^*V600E*^ (13% *vs*. 1%, *P* = .04) mutations might benefit higher percentages of young females than old patients (Figure S2b). Of note, no young male CCA patients were identified with *FGFR2* fusions or *BRAF*^*V600E*^ mutations, and *ERBB2* and *BRCA* actionable mutations were exclusively observed in the old subgroup. Although *KRAS* mutations were more frequently detected in old patients than in young patients, the proportions of *KRAS*^*G12C*^ inhibitors eligible patients were close between two subgroups (2% *vs*. 4%, *P* = .51).

## Discussion

We addressed a comprehensive comparison of molecular features, prognosis data, and actionable mutations for target treatment between young and old CCA patients. Compared to 25 young patients whose CCA development mechanism was probably related to alcoholism consumption, CCA might attribute to DNA repair issues or nonalcoholic fatty liver in 163 old patients. Although no significant difference in OS was observed between young and old CCA patients, *KRAS* and *TERT* promoter mutations, which displayed higher prevalence in old patients, were associated with inferior OS. Nevertheless, compared to old patients, the proportion of patients who were potentially eligible for target treatment was likely to be larger in young patients, especially young females, due to more common *FGFR2* fusions and *BRAF* mutations.

Previous studies have not drawn consistent conclusions on whether old CCA patients have poorer prognosis than young patients. Based on 11,127 iCCA patients enrolled between 1995 and 2004, higher 1-year (HR: 1.77, 95% CI: 1.56–2.02) and 5-year all-cause mortalities (HR: 1.65, 95% CI: 1.49–1.84) were observed in patients at least 45 years old, compared to patients below 45.^11^ Age was also identified as a separate factor predicting prognosis among gallbladder cancer and iCCA patients proceed with gemcitabine and S−1 combination chemotherapy as first-line palliative treatment.^32^ Similarly, patient age could serve as a prognostic factor for hilar CCA patients treated with surgical resection.^33^ In present study, we did not detect a significantly inferior OS among old patients, which was consistent with a couple of previous studies. For instance, in one study including 136 CCA patients, OS was not strongly associated with patient age (HR: 1.01, 95% CI: 0.99–1.03).^34^ Another study focused on CCA patients treated with resection neither detected an obvious relationship between prognosis and age.^35^ Of note, in a part of previous studies, patient age was treated as a numeric variable and included in regression models directly; however, in other studies where patient age was used as a categorical variable, different thresholds were applied over patient age to identify young and old patients. Moreover, the CCA subtype compositions differed across studies, and several studies focused on BTC also included patients with gallbladder cancers. Therefore, the heterogeneity of age group threshold and subtype of CCA cases might result in inconsistent conclusions on the association of patient age with prognosis.

In our study, *KRAS* and *TERT* alterations were enriched in old patients over 45 and inferior OS was associated with altered *KRAS* or *TERT* gene, which could partially explain the worse OS in old patients observed in some previous studies. One study where 81% of 195 total patients were diagnosed with iCCA also demonstrated that altered *KRAS* gene, detected in 13% patients, was negatively related to OS (*P* = .026). That study also showed *KRAS* alterations occurred with greater frequency in eCCA; however, the enrichment of *KRAS* alterations in old patients was unable to be confirmed due to lacking relevant data.^16^ In another study including 85 (85/123, 69%) iCCA patients and investigating ctDNA from their blood samples, a trend of higher prevalence of altered *KRAS* genes among patients ≥50 years was observed, even though the difference between early-onset (age <50 years) and old patients was not statistically significant (approximately 33% *vs*. 21%).^36^ Conversely, *TERT* promoter mutations appeared to be more commonly detected in early-onset patients than old patients (approximately 10% *vs*. 2%). In comparison with early-onset patient, *TP53* mutation prevalence was relatively high in old patients (67% *vs*. 35%). Although we did not observe such an enrichment of mutated *TP53* genes in the old subgroup of our study cohort, a strong association between *TP53* mutations and poorer OS was observed. Similar negative association between *TP53* mutation and clinical outcomes was also observed in BTC patients undergoing surgery treatment^37^ We demonstrate, for the first time to our knowledge, the potentially distinctive prognosis between young and old CCA patients might be rationalized by different prevalence of genetic alterations, whereas larger study cohorts with diverse ethnic groups are required to further confirm our findings.

Development of drugs targeting genetic alterations in CCA has got great achievements since the genomic profiling studies being launched. Ivosidenib is one potential option for iCCA patients, of whom *IDH1/2* mutations are frequently identified, after chemotherapy-refractory. In one phase III randomized controlled trial including 185 post-chemotherapy iCCA patients, significantly improved PFS was observed in patients receiving ivosidenib, in comparison with placebo (HR: 0.37, 95% CI: 0.25–0.54).^22^ For patients harboring *FGFR1/2/3* fusion, detected in approximately 5% patients in our study cohort, several *FGFR* inhibitors have been developed.^20,38^ Also, the combination of *BRAF* and *MEK* inhibitors, dabrafenib plus trametinib, showed promising treatment effects among CCA patients carrying *BRAF*^*V600E*^ mutations.^39^ However, owning to the *FGFR2* fusion less commonly identified among fluke-related CCA patients.^40^ as well as the low prevalence of *BRAF*^*V600E*^ mutations, targeted therapy plans should be developed depending on personal genomic profiles. Moreover, over 10% patients in our study cohort harboring *BRCA1/2* mutations, *ERBB2* or *MET* CNV gain, for which potential targeted therapy might be available. It was worth noting that only approximately 10% altered *KRAS* genes were *KRAS*^*G12C*^ and most *KRAS*^*G12X*^ mutations were other than *KRAS*^*G12C*^, suggesting the studies in which the efficacy of *KRAS*^*G12C*^ inhibitors could be investigated in extended cohorts. Old patients might get great benefit from these studies, as they were observed with higher *KRAS* mutation prevalence than young patients. For immunotherapy, a group of studies have indicated the modest efficacy in CCA, and a phase III clinical trial of pembrolizumab combined with gemcitabine and cisplatin is ongoing.^41^ Although chemotherapy is currently serving as the standard of care for CCA patients not eligible for surgical resection, personalized targeted therapy and immunotherapy guided by genomic profiles exhibited improved clinical outcomes when compared to routine chemotherapy (objective response rate: 87.5% *vs*. 25%, *P* < .001; mOS: not reached *vs*. 6.5 months, HR: 0.10, 95% CI: 0.02–0.48).^42^

Our study does have limitations, mainly including unknown CCA subtypes, and considerable missing data of treatment history. As a result, we were neither able to conduct stratified analyses for each CCA subtype separately nor to provide a landscape view of CCA management in real world. Another limitation was the lack of clinical stages at initial diagnosis, leading to the difficulty of controlling for the potentially confounding effect of clinical stages on OS. We had no prognosis OS data in our own study cohort, and main findings in prognosis was based on the external cohort mainly consisting of CCA cases in western countries. However, genomic landscape and etiology of CCA might vary in different countries, such as the higher proportion of microsatellite instability-high CCA patients in Asian countries,^43^ resulting in potentially limited generalizability of the conclusion in prognosis. Additionally, due to the lack of clinical data, we were neither able to show Carbohydrate Antigen 19–9 levels of our study cohort nor to compare the difference between young and old patients. Finally, we could not assess hepatitis B/C virus infection history because of the retrospective structure of our study, and the sample size of young patients below 45 years was relatively small. Further studies with more young patients and complete hepatitis data were needed.

## Conclusions

Previously reported inferior prognosis in old CCA patients might be rationalized by more frequent *KRAS*/*TERT* alterations related to poor OS. Over 35% patients were suitable for potential personalized targeted therapy or immunotherapy, and young females were more likely to be eligible for treatment targeting *FGFR* fusions or *BRAF*^*V600E*^ mutations.

## Data Availability

The datasets used and/or analyzed during the current study are available from the corresponding author on reasonable request.

## Abbreviations

BTC: biliary tract carcinoma
CCA: cholangiocarcinoma
CI: confidence interval
CNV: copy number variation
dCCA: distal cholangiocarcinoma
eCCA: extrahepatic cholangiocarcinoma
FFPE: formalin-fixed paraffin-embedded
HCC: hepatocellular carcinoma
HR: hazard ratio
iCCA: intrahepatic cholangiocarcinoma
mOS: median overall survival
mRFS: median relapse-free survival
NGS: next-generation sequencing
OS: overall survival
pCCA: perihilar cholangiocarcinoma
PFS: progression-free survival
TMB: tumor mutational burden
